# Computer Implementation of a New Therapeutic Model for GBM Tumor

**DOI:** 10.1155/2014/481935

**Published:** 2014-08-05

**Authors:** Ali Jamali Nazari, Dariush Sardari, Ahmad Reza Vali, Keivan Maghooli

**Affiliations:** ^1^Department of Medical Radiation Engineering, Islamic Azad University, Tehran Science and Research Branch, Tehran 14515-775, Iran; ^2^Electrical Engineering Department, Control Engineering Group, Malek Ashtar University of Technology, Tehran, Iran; ^3^Department of Biomedical Engineering, Islamic Azad University, Science and Research Branch, Tehran, Iran

## Abstract

Modeling the tumor behavior in the host organ as function of time and radiation dose has been a major study in the previous decades. Here the effort in estimation of cancerous and normal cell proliferation and growth in glioblastoma multiform (GBM) tumor is presented. This paper introduces a new mathematical model in the form of differential equation of tumor growth. The model contains dose delivery amount in the treatment scheme as an input term. It also can be utilized to optimize the treatment process in order to increase the patient survival period. Gene expression programming (GEP) as a new concept is used for estimating this model. The LQ model has also been applied to GEP as an initial value, causing acceleration and improvement of the algorithm estimation. The model shows the number of the tumor and normal brain cells during the treatment process using the status of normal and cancerous cells in the initiation of treatment, the timing and amount of dose delivery to the patient, and a coefficient that describes the brain condition. A critical level is defined for normal cell when the patient's death occurs. In the end the model has been verified by clinical data obtained from previous accepted formulae and some of our experimental resources. The proposed model helps to predict tumor growth during treatment process in which further treatment processes can be controlled.

## 1. Introduction

Human brain is composed of cells called glios that proliferate to repair its injured part. This reproduction is totally under control of the brain. However, if any of these cells, without brain control, starts doubling for any reason, it will generate an unwanted tumor. GBM or glioblastoma multiform is the most malignant and aggressive type of high-grade gliomas which incur major clinical problems and mostly leads to death.

There are two types of brain cancer. Primary tumors are those that originate from the brain itself. The second type originates from metastasis in other parts of patient's body. The primary tumors may become cancerous or not; however, it needs room to grow; therefore, some types of injuries occur. Despite many advancements in surgery, radiotherapy, and other treatment methods, no effective treatment have been proposed in the previous decades and most of patients die of this type of cancer. Thus, primary brain tumors bring high mortality rate, which is attributed to short doubling time of the tumor. The treatment of this tumor encounters a series of problems. The tumor does not respond to schemes of treatment as expected. Another factor is related to the treatment plans that are not that much impressive and finally in major number of cases mathematical modeling is not used to provide an optimal dose delivery to the injured tissue.

Radiotherapy, being one of the main clinical solutions for cancer treatment, is technically the second remarkable method after surgery. Widespread application of this method is due to several factors like high precision, fast response, high-tech apparatus, and many decades of knowledge and experience. However, there are some disadvantages. Sometimes normal tissues are inevitably hit by radiation beams during dose delivery to the cancerous tissues. In order to gain a better view of injuries to normal and cancerous cells, a mathematical model is required. In the present paper, a new model of GBM tumor has been generated that does not suffer from deficiencies existing in other models.

GBM or glioblastoma multiform is the most malignant and aggressive type of high-grade gliomas which follow major clinical problems and mostly in 10 to 12 months leads to death [1]. Harpold et al. introduced [2] a model with radiotherapy input which was improved at the same year. Consequently a continuum mathematical model about the invasive cell treatment was developed [3]. A remarkable masterpiece of work was created for the first time in 2007 [4] which followed a two-state space equation without considering the input term of radiotherapy or chemotherapy. Tanaka et al. [5] worked out a hybrid model that showed that the proliferation of cancerous cells is dependent on the radius of the tumor. In 2010 [6] a model with the input term was written that used the radiobiological aspects. Based on this achievement, Barazzuol et al. [7] pushed the model further, considering the radiotherapy and chemotherapy but in a complicated way. Specifications of this model are investigated in the next section.

## 2. Model Development

Detailed information about properties and disadvantageous of abovementioned models is investigated.

Tanaka and his colleagues [5] suggested a hybrid compartment-continuum-discrete (CCD) model in order to simulate the proliferation of gliomas and the cell invasion. It is described by the equation below:

(1) $$\begin{array}{c} C\left(r\right)=C_{0}-\left(\frac{s}{6D}\right)\left(R^{2}-r^{2}\right)K_{l}\left(\frac{1}{r}-\frac{1}{R_{T}}\right). \end{array}$$

One deficiency of this model is lack of state space equations and thus control over the system (tumor growth) is not easy. The other negative point is that the time parameter is missing and the future status of the cells cannot be estimated.

Harpold et al. have written a review over previous models and tried to cast the proliferation of gliomas in a model. Their final result is model appearing in (2). Outcomes have slight difference with the aforementioned model [2].

Consider

(2) $$\begin{array}{c} \frac{\partial c}{\partial t}=\;\nabla \cdot \left(D\nabla c\right)+\rho c-G\left(t\right)c. \end{array}$$

Harpold and his team continue their work and in 2007 their new model that has the net proliferation of gliomas cells [2] is

(3) $$\begin{array}{c} \frac{\partial c}{\partial t}=\;\left(D\nabla^{2}c\right)+\;\rho c\left(1-\frac{c}{k}\right), \end{array}$$

where ∂*c*/∂*t* is the rate of change of gliomas cell concentration and (*D*∇^2^ *c*) is the net invasion of glioma cells and *ρc*(1 − (*c*/*k*)) is the net proliferation of gliomas cells.

It is observed that the model contains time parameter and estimation of the amount of cancer cells is accessible but the disadvantage is the difference between mathematical results and experimental data. Besides, the status of normal cells is not simultaneously investigated and the death moment is not computable.

Stein et al. [3], in the same year in a study with the aim of defining the invasive cells treatment in glioblastoma, used a mathematical model called “continuum mathematical model” which is as follows:

(4) χ2(D,vi,s,g) =1N−n−1[∑t(Ri(t)−  Rˇi(t)σR)2       +∑r(ui(r,t3)−uˇi(r,t3)σu)2].

Its disadvantages include using too many parameters that have negligible effects on the final result plus the deficiencies of former models.

Another work we want to mention is about what Rockne and his team did in 2008 [8]. They used the Swanson model and added the radiobiological effect by using a *R*(*x*, *t*)*c* term to the basic model of Swanson. They proposed the below model in a way that the LQ model of radiobiology is a part of their work:

(5) $$\begin{array}{c} \frac{\partial c}{\partial t}=\nabla \cdot \left(D\left(x\right)\nabla c\right)+\rho c-R\left(x,t\right)c, \end{array}$$

where ∂*c*/∂*t* is the rate of change of gliomas cell concentration, ∇·(*D*(*x*)∇*c*) is the net dispersal of gliomas cells, *ρc* is the net proliferation of gliomas cells, and *R*(*x*, *t*)*c* is the loss due to therapy.

Later in 2010 in a study with 9 patients [6] in whom the tumor had been diagnosed soon enough and underwent radiotherapy treatment, their model was improved on the basis of radiobiology of each patient for the increase of GBM cells. This model contains the LQ model of radiology in a more effective manner. This model responds to the treatment and for the first time the GBM model was combined with dose delivery input, which makes further investigation on the type of treatment schemes possible.

Consider

(6) ∂c∂t=∇·(D(x)∇c)+ρc(1−ck) −R(x,t,Dose)c(1−ck).

The first three terms are the same as above and the last term  *R*(*x*, *t*)*c* is modified as follows:

(7) R(x,t,Dose(x,t)) ≡{0for  t∉therapy(1−S(α,β,Dose(x,t)))for  t∈therapy}.

To obtain a general view of disadvantages of existing models, we can put forward points as using too many parameters that have negligible effects on its final result. The models' mathematics is not that much precise and is based on experimental data. The time parameter is missing and so estimating the future status of the cells is not available. The status of normal cells is not simultaneously investigated and the decease time of the patient is not computable. Most of papers here just have many statistics and they lack computation.

In our model we provide new inputs in which the above deficiencies will be resolved. There are also novel issues in the proposed model.

## 3. Methodology

In the previous section, the deficiencies of former model have been declared. The present model consists of new parts that compensate most of the disadvantages of the other existing works.

What Rockne et al. [6] and Barazzuol et al. [7] did in 2010 is the basis of our idea. Their work is really effective and describes the status of normal and cancerous cell through time, but still its equations have just initial condition and lack the specific input. Here is their formula in two equations

(8) $$\begin{array}{c} \frac{dN}{dt}=-k_{n}\cdot N\cdot C,\\ \frac{dC}{dt}=k_{c}\cdot C. \end{array}$$

*N* is the number of normal cells and *C* is the cancerous ones. The other parameters are defined in the nomenclature part of this paper. Obviously, one can see that the states of *N* and *C* are not related to radiation dose input.

Although many of the other model deficiencies, like dependency on time, the stats of normal cells, and state space modeling, have been considered in this model, still there are two main drawbacks in this model. It has not been associated with an input term of radiotherapy. The effectiveness of dose for normal and cancerous cells is not defined. Barazzuol and other authors in 2010 [7] mentioned that according to the LQ model (see Appendix section) each cell (normal or cancerous) is diminished facing the treatment radiation and the main goal of this model is to open a new pathway for control issues. Since the Kirkby model lacks an input term, the amount of the interval of the treatment scheme for the GBM patients is not under control. If the input dose of radiation through this novel model is mathematically defined instead of equal nonprecisely calculated pulses of radiation a highly variable source of the decision making will be available for technical scientists to investigate ideas of their own about generating new plans in GBM treatment schemes.

There are four series of experimental data set that helped us in the GEP algorithm to generate the proposed model. In each set of data a specific portion of dose is delivered to the patients and various results have been accessed and are shown in Tables 2 and 3. It is easily seen that hyperfractionation (dose delivery is fractionated in more than once a day during treatment) affects the proliferation of normal and cancer cells in a way that death occurs after a longer period which prolongs lifetime of patient.

In the first set of data as can be seen in Table 2 the whole treatment dose is delivered consistently. The second table shows a patient data that received the treatment in four parts of hyperfractionation, which obviously lead to a longer lifetime for the patient. Gene expression programming (GEP) similar to genetic algorithms (GA) and genetic programming (GP) is a gene based algorithm. Firstly by using populations of individuals, it selects them considering their fitness and introduces genetic variation using one or more genetic operators [9].

GEP uses chromosome's character linearly organized in a head and a tail made of genes. By means of mutation, the chromosome functions as a genome and is exposed to modification, transposition, root transposition, gene transposition, gene recombination, and one- and two-point recombination [10].

In the beginning (7) should be combined with LQ model (see Appendix section) in order to obtain a new differential equation which indicates the number of cancerous and normal cells of brain in the injured area. The equation should manifest the dose amount and the interval of each portion of dose delivery simultaneously. As it is said before (8) with a slight change is as follows:

(9) $$\begin{array}{c} \frac{dN\left(t\right)}{dt}=-k_{n}\cdot N\left(t\right)\cdot C\left(t\right),\\ \frac{dC\left(t\right)}{dt}=k_{c}\cdot C\left(t\right). \end{array}$$

In the first step the above equation must be digitized and converted to a discrete form like this

(10) $$\begin{array}{c} N_{i}=-\Delta t\left(k_{n}*N*C\right)+N_{i-1},\\ C_{i}=\Delta t\left(k_{c}*C\right)+C_{i-1}. \end{array}$$

Based on (2) and the LQ equation (see Appendix section), the above information is given to the regression equation of GEP as the input or initial function.

The objective of the present work is the discovery of a symbolic expression that satisfies a set of fitness cases. First, the set of functions *F* and the set of terminals *T* must be chosen [10].

Based on the above statements the input functions of the GEP algorithm are defined as follows. The four main operations as +, −, ∗, and / and ∧, also exponential functions like *ex* and ln⁡⁡(*x*), the triangular functions sin⁡(*x*), cos⁡⁡(*x*), and tan⁡(*x*) and hyperbolic functions sinh⁡⁡(*x*), cosh⁡⁡(*x*), and tanh⁡(*x*) and sin − 1⁡(*x*), cos⁡−1⁡(*x*), and tan − 1⁡(*x*) and SQRT are chosen for the GEP equation. The length of head and tail in the algorithm is 15 and 16, respectively. The number of initial sample is supposed to be 512. Therefore, the total amount of operations and operands are 31 (15 + 16). It is seen that 18 functions are proposed for this problem and there are 5 separate inputs are introduced which are

1. the initial condition that is the number of normal and cancerous cells (*N*_0_ & *C*_0_) in the first day of treatment and before treatment starts;
2. the time intervals of dose delivery Δ*t*: this parameter defines the amount of time that should be considered for each fraction of treatment, for instance, once a day, twice a day, and so forth;
3. the amount of dose in each fraction of therapy;
4. the amount of average absorbed dose and its effect over normal and cancerous cells in each individual patient in accordance with its physical condition.

In the algorithm utilized in our work, there are 18 operations (mathematical functions) and 5 operands (variables), summing up to 23. The nearest number to 23 in a binary format GEP is 32 which is 25. The length of the matrix based on 2 is 5 × 31 or 155 given the reason that 31 is the summation of the whole set. The initial number is 512, so the total dimension of the data is 155 × 512. A map for converting a digit more than 23 to a number between 1 to 23 is required here, since the operations such as mutation and crossover provide a digit more than the 23, the mutation probability is 0.1, and the crossover is supposed to be 13. The model is then capable of estimating the future status of the patient's treatment, if the proposed data, (8), and LQ model according to clinical dataset are combined by the GEP algorithm. The least square method (LSM) is then applied to choose the best fitting model gained from the GEP.

The chosen sample is used for the regression and the obtained function is tested. There have been 100 sets of sample data divided into 4 groups. The first category has its dose delivery once a day.

The second one has its dose delivery twice a day and subsequently the third one three times a day and the last one is three times a day but with nonequal amount of dose.

Among each of 25 samples data, the first 20 have been used for training and the remaining 5 for testing. Since in each set of 25 samples data 7 to 10 of them treat totally different of the proposed model, data mining and PCA algorithm are applied to eliminate them from the training and test process. At the end the calculated model by using (7) and LQ model in their best form is suggested as follows:

(11) Ni+1=−kn·Ni·Ci·dr−(1−doseEff)·kk2·dt ×(1−exp⁡⁡(−αd−βd2))Ni+Ni,Ci+1=1+dt·kc−tt·kk2·dt·doseEff ·(1−exp⁡⁡(−αd−βd2))Ci.

If the relations achieved by GEP are converted to a differential equation the following is generated:

(12) dNdt=−kn·N·C−(1−doseEff) ·kk2·(1−e−α.d−βd2)N,dCdt=kc·C−tt·(doseEff)·kk2·(1−e−α·d−βd2)C.

The formula parameters are as follows in Table 1.

In one portion of dose delivery each day the amount of dose was 1 Gy which was reduced to 0.5 Gy in the next categories.

## 4. Model Verification

In order to verify this model, a set of clinical data is required. Previous articles and our experimental tests provide this most important part of the present work. The model is fitted to attained data and the Kaplan-Meier [9] survival curves have been used. The weighting was adjusted heuristically to guide the optimization to fit correctly the tail of the curve representing the long-term survivors.

## 5. Results

Tables 2 and 3 are the tables of dose delivery for two separate patients as a sample of the clinical data. Table 2 is related to a patient that received 1 Gy of dose once a day.  Table 3 is the data of another patient who had 0.5 Gy of radiation dose 4 times a day in different scheme of treatment.

**Figure 1:**
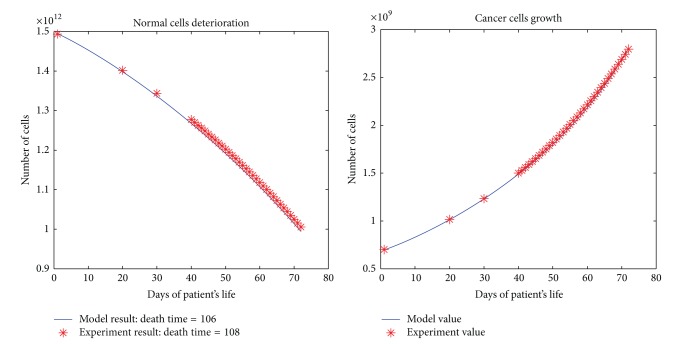
The cancerous and normal cells model and experimental result, death time, and total dose of a patient with one time per day dose delivery.

**Figure 2:**
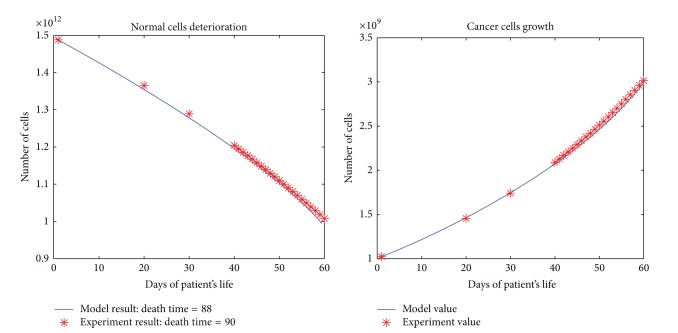
The cancerous and normal cells model and experimental result, death time, and total dose of a patient with two times per day dose delivery.

**Figure 3:**
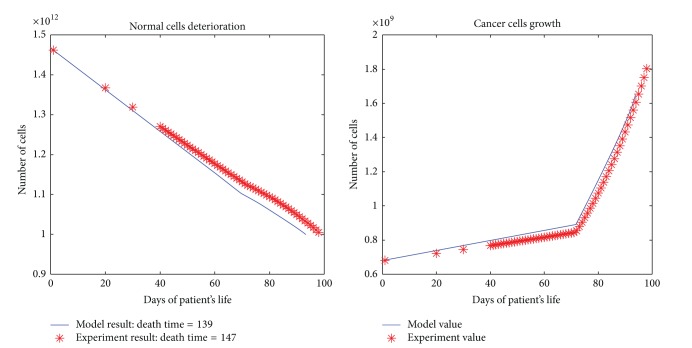
The cancerous and normal cells model and experimental result, death time, and total dose of a patient with three times per day dose delivery.

**Figure 4:**
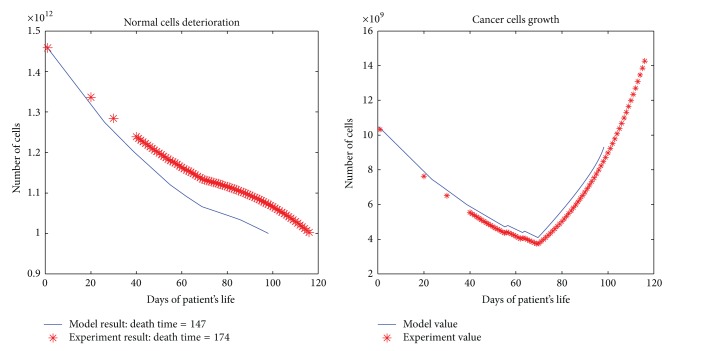
The cancerous and normal cells model and experimental result, death time, and total dose of a patient with four times per day dose delivery.

**Figure 5:**
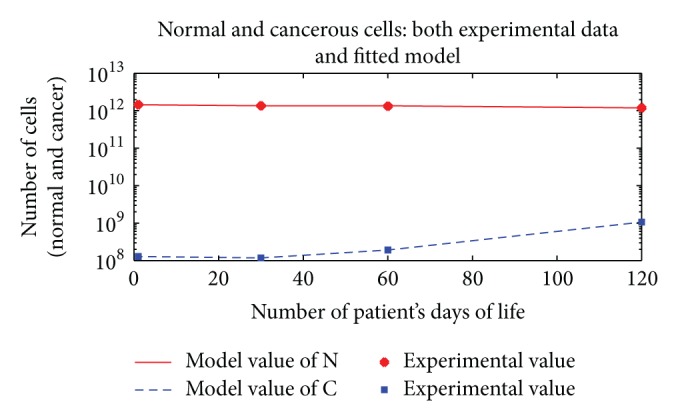
The growth of cancer cells in comparison with decay of normal brain cells.

All the results are depicted in Figures 1, 2, 3, 4, and 5. Each figure compares the error between the model response and the clinical data.

## 6. Conclusion

It is easily seen that this new model can efficiently empower the radiation oncologist and medical physicist control over the treatment procedure in comparison with the traditional models. Since new features according to the clinical and experimental data have been added to the proposed model, better and more reliable results have been generated. Here are some suggestions.

Increase in the number of clinical data can lead to more precise results. This investigation experienced around 95.2 percent of accuracy while fitting the theoretical data on the clinical ones. This value is obtained by using the average of error between each point of clinical and mathematical data.

We use a few variable parameters in this model, but one can obtain better formulae using a set of other parameters which are effective in cancer growth and modification.

Finally, the best further work is to generate a new intelligent algorithm to have new schemes of radiotherapy in which constant dose delivery is eliminated and new functions of dose are added for each individual patient. It can lead to a longer lifetime for the patient and easily estimating the remaining length of patients' lives.

## Figures and Tables

**Table 1:** Important parameters in the model.

*d*	Dose delivery amount
*α* and *β*	Radiobiological ratios *β* = *α*/9
*K*_*N*_, *n*, *c*, *K*_*c*_	The parameters of (8)
*kk2* = 2*K*_*c*_	Proportional coefficient made by GEP
*tt*	1 in normal dose delivery and 1.15 for twice a day
doseEff	The effectiveness percent of dose delivery to *N* and *C* cells

**Table 2:** A patient that received 1 Gy of dose once a day.

Day number (*t*)	*N*(*t*)-experiment	*N*(*t*)-model	*C*(*t*)-model	*C*(*t*)-experimental		
1	1.46*E* + 12	1.4764*E* + 12	1.06*E* + 9	1.08*E* + 9	*C*(0): initial cancer cells	Almost 1*E* + 8
17	1.35*E* + 12	1.33264*E* + 12	1.45*E* + 9	1.43*E* + 9	*K*_*n*_	3.35*E* − 12
22	1.31*E* + 12	1.30808*E* + 12	1.6*E* + 9	1.64*E* + 9	*K*_*c*_ = ln⁡(2)/*T*_*D*_	0.0288
32	1.23*E* + 12	1.20807*E* + 12	1.94*E* + 9	1.74*E* + 9	Alpha	0.17
38	1.17*E* + 12	1.16807*E* + 12	2.18*E* + 9	2.28*E* + 9	Dose_eff	0.93
44	1.12*E* + 12	1.16803*E* + 12	2.45*E* + 9	2.44*E* + 9	Beta	0.02
47	1.08*E* + 12	1.16784*E* + 12	2.59*E* + 9	2.68*E* + 9	Number of doses per day	1
51	1.04*E* + 12	1.16678*E* + 12	2.8*E* + 9	2.84*E* + 9	Death time	56th day
53	1.02*E* + 12	1.161*E* + 12	2.91*E* + 9	2.92*E* + 9	*α*/*β*	*≅*9 : (0.17/0.02)
56	9.92*E* + 11	1.12971*E* + 12	3.09*E* + 9	3.09*E* + 9		

**Table 3:** A patient that received 0.5 Gy of dose 4 times a day.

Day number (*t*)	*N*(*t*)-experimental	*N*(*t*)-model	*C*(*t*)-model	*C*(*t*)-experimental		
1	1.45*E* + 12	1.46942*E* + 12	4.35*E* + 8	4.45*E* + 8	*C*(0): initial cancer cells	Almost 1*E* + 8
22	1.34*E* + 12	1.33188*E* + 12	3.21*E* + 8	3.41*E* + 8	*K*_*n*_	3.35*E* − 12
44	1.25*E* + 12	1.25188*E* + 12	2.33*E* + 8	2.45*E* + 8	*K*_*c*_ = ln⁡(2)/T_D_	0.0288
53	1.21*E* + 12	1.22488*E* + 12	1.95*E* + 8	1.98*E* + 8	Alpha	0.17
70	1.16*E* + 12	1.18184*E* + 12	1.64*E* + 8	1.55*E* + 8	Dose_eff	0.93
92	1.13*E* + 12	1.15316*E* + 12	3.10*E* + 8	3.22*E* + 8	Beta	0.02
111	1.10*E* + 12	1.11031*E* + 12	5.37*E* + 8	5.38*E* + 8	Number of doses per day	4
125	1.07*E* + 12	1.09309*E* + 12	8.04*E* + 8	8.03*E* + 8	Death time	143th day
135	1.03*E* + 12	1.02347*E* + 12	1.07*E* + 9	1.09*E* + 9	*α*/*β*	≅9 : (0.17/0.02)
143	9.99*E* + 11	9.97391*E* + 11	1.35*E* + 9	1.41*E* + 9		

## Appendix

### The LQ Model

Consider

(A.1) $$\begin{array}{c} E=\alpha \cdot \text{Dose}+\beta {\text{Dose}}^{2},\\ S=e^{-E}. \end{array}$$

## The Nomenclature

*C*(*t*): Number of brain cancer cells
*C*_0_: Initial number of cancer cells in brain at its presentation (cells)
*K*_*c*_: Rate constant for cancer cell growth (days_1) *K*_*c*_ is related to the doubling time (*T*_*D*_)
*K*_*n*_: Rate constant for normal cell damage and deterioration (cells_1 days_1)
*N*(*t*): Number of normal brain cells (cells)
*N*death: Critical number of normal brain cells required
*t*_*D*_: Tumor doubling time (days) ln2/ *K*_*c*_
*N*_0_: Number of normal brain cells left at presentation (cells)
*t* and *tt*: Time (days) tumor age at presentation (days)
*d*: Dose
*kk*2: A proportional coefficient
doseEff: The effect of dose on normal and cancer cells.

## References

[1] Alvord EC, Shaw CM, Duckett S (1991). Neoplasms affecting the nervous systems in the elderly. *The Pathology of the Aging Human Nervous System*.

[2] Harpold HLP, Alvord EC, Swanson KR (2007). The evolution of mathematical modeling of glioma proliferation and invasion. *Journal of Neuropathology and Experimental Neurology*.

[3] Stein AM, Demuth T, Mobley D, Berens M, Sander LM (2007). A mathematical model of glioblastoma tumor spheroid invasion in a three-dimensional in vitro experiment. *Biophysical Journal*.

[4] Kirkby NF, Jefferies SJ, Jena RA (2007). A mathematical model of the treatment and survival of patients with high-grade brain tumours. *Journal of Theoretical Biology*.

[5] Tanaka ML, Debinski W, Puri IK (2009). Hybrid mathematical model of glioma progression. *Cell Proliferation*.

[6] Rockne R, Rockhill JK, Mrugala M (2010). Predicting the efficacy of radiotherapy in individual glioblastoma patients in vivo: a mathematical modeling approach. *Physics in Medicine and Biology*.

[7] Barazzuol L, Burnet NG, Jena R, Jones B, Jefferies SJ, Kirkby NF (2010). A mathematical model of brain tumour response to radiotherapy and chemotherapy considering radiobiological aspects. *Journal of Theoretical Biology*.

[8] Rockne R, Alvord EC, Szeto M, Gu S, Chakraborty G, Swanson KR (2008). Modeling diffusely invading brain tumors an individualized approach to quantifying glioma evolution and response to therapy. *Selected Topics in Cancer Modeling*.

[9] Mitchell M (1996). *An Introduction to Genetic Algorithms*.

[10] Ferreira C (2001). Gene expression programming: a new adaptive algorithm for solving problems. *Complex Systems*.

